# Low Daily Intake of Fruits and Vegetables in Rural and Urban Bangladesh: Influence of Socioeconomic and Demographic Factors, Social Food Beliefs and Behavioural Practices

**DOI:** 10.3390/nu13082808

**Published:** 2021-08-16

**Authors:** Sadia Mustafa, C. Emdad Haque, Soham Baksi

**Affiliations:** 1 Natural Resources Institute, University of Manitoba, Winnipeg, MB R3T 2N2, Canada; mustafa3@myumanitoba.ca; 2 Department of Economics, University of Winnipeg, Winnipeg, MB R3B 2E9, Canada; s.baksi@uwinnipeg.ca

**Keywords:** sociodemographic factors, low fruit and vegetable intake, beliefs and behavioural practices, Bangladesh

## Abstract

Bangladesh is facing a large burden of non-communicable diseases. As a possible remedy, the WHO/FAO recommends consuming 400 g or five servings of fruits and vegetables every day; however, only a small proportion of the population practices this. The present study sets out to determine the sociodemographic factors that affect this low intake of fruits and vegetables, and the roles that beliefs and behavioural practices play in influencing food consumption. Logistic and ordered logistic regressions were used to identify what sociodemographic factors are significantly influencing fruit and vegetable intake, and to explain the role of social food beliefs. It was found that in Bangladesh 75% of urban and 92% of rural populations consume less than five servings a day. While gender was not found to be a significant factor, housewives appeared to be more at risk of a lower intake of fruits and vegetables. People with higher income, higher education, and who are older were all less likely to have problems with a low intake of fruits and vegetables. Higher education assisted in attaining positive beliefs and behavioural practices regarding food, while residing in a rural community was found to be a significant constraint.

## 1. Introduction

Around 41 million people die each year globally because of non-communicable diseases (NCDs), 77% of which occur in low and middle income countries (LMICs) [1]. Numerous studies reveal that regular intake of adequate quantities of fruits and vegetables contribute to improved health and can create immunity against NCDs [2]. Food choices cannot include everything edible, but need to be healthy and should contain fruits and vegetables. In light of such findings, the WHO/FAO recommends a minimum 400 g (or five servings) of fruits and vegetables every day to curb NCDs [3]. Worldwide, emphasis on fruit and vegetable consumption has increased noticeably in recent decades as part of efforts to reduce the number of deaths from NCDs.

Despite these recommendations and promotion by international agencies, people in many LMICs consume far less than the required five servings. For example, studies in Iran, Kenya and Tanzania respectively observed that 87.5%, 94% and 82% of adults eat less than five servings a day [4,5,6]. However, no significant difference in fruit and vegetable consumption was observed between Iranian urban and rural residents. Research in Tanzania, Iran and Thailand found that rural inhabitants, the young and single, those with no income, people with high tobacco use and men were all more likely to have inadequate fruit and vegetable intake [4,6,7]. Urban Kenyan women who were less educated were also found to be at higher risk of inadequate fruit and vegetable intake [6]. As occupational category often matters in fruit and vegetable consumption, Thai people who worked in agriculture were more likely to eat more fruits and vegetables than those who owned businesses or worked as manual labourers in a private firm or a government agency [7]. Income is also an important determinant of fruit and vegetable intake; expenditure analysis in poor communities in South Africa showed spending $71.4 and more on food monthly and having a private vehicle increased the likelihood respectively to 1.6 and 2.1 times more to consume two or more servings of fruits and vegetables than those who spent less on food or used public transport to go to stores [8].

It is obvious that hunger is the key driver for eating. However, dietary choices and preferences are not driven only by biological and nutritional needs [9], economic, physical, social and psychological components also play considerable roles in determining food choices [10]. Although numerous studies have explored the social determinants of healthy diets among people in high income countries [11], research on the determinants of healthy food choices in LMICs is still scarce [12]. For example, there are various social, geographic and cultural norms that people follow in considering food consumption. In general, the literature on what sociodemographic characteristics influence food knowledge, social beliefs and behavioural practices is scant globally, and is even rarer in the LMIC context. One such study, involving female Sudanese students at Ahfad University, found that most of them lacked adequate knowledge of nutrition, and 42.4% gained what knowledge they had from mass media [13]. Another rare example of this research in the LMIC context, in South Africa, revealed that neither a positive attitude towards healthy eating nor level of education were associated with inadequate eating [8].

The kinds of attitudes that may influence eating behaviours has received some attention in the literature. The most popular models in this area of study are the Attitude-Social Influence-Efficacy and the Planned Behaviour models [14,15]. As identified by Brug et al. [16], three types of psychological factors tend to influence dietary-related behaviour: (i) attitudes, (ii) social influence and (iii) self-efficacy. Here, *attitude* is related to whether a person holds positive or negative outlook about fruit and vegetable intake; *social influence* refers to the conditions and supports in the surroundings in favour of or opposed to this particular eating behaviour; and *self-efficacy* implies the ability of a person to perform the desired behaviour. In many LMICs, food beliefs and taboos are prevalent and influence peoples’ attitudes from generation to generation, mostly about appropriate eating during pregnancy and physical illness [16]. Another belief is regarding the social status of crops: low status (mostly indigenous) crops are often called ‘poor man’s crops’, while high status, expensive crops are considered ‘rich man’s crops’ [17,18].

The above overview of correlates of low fruit and vegetable consumption in various LMICs reveals several key sociodemographic and attitudinal factors. It is apparent that most correlates are related to gender, residence, age, income, occupation and lifestyle. Like most LMICs, the population of Bangladesh is burdened with a very high proportion NCDs and associated fatalities. To develop effective interventions and further promote the consumption of fruits and vegetables in Bangladesh, a better understanding of the factors driving the low consumption of fruits and vegetables and the processes associated with taboos and beliefs influencing food choices is urgently needed.

In consideration of this backdrop, the specific objectives of this paper are, in relation to rural and urban Bangladesh to determine the sociodemographic factors affecting (i) low intake of fruits and vegetables, and (ii) the social beliefs and behavioural practices concerning low fruit and vegetable intake.

## 2. Methods and Materials

### 2.1. Study Area and Sampling

The study area in Bangladesh is divided into rural and urban components. In this country, 70% of the land (mainly rural) is devoted to agriculture [19]. Rural population consists of 62% of the total population whereas the remaining (i.e., 38%) lives in the cities [20]. In Bangladesh, the culture of collective living (joint family) and farming with a patriarchal rule of descent are prevalent. This is reflected in that only 15.8% of the households are female-headed [21]. Collective or joint families consist of at least three generations (grandparent, parent, children) and the head of the household is usually the eldest male. Only in rare cases the household heads are the eldest females. As a result, the mean age of the household heads is generally higher than the younger age cohort of 25–35. These demographic features of the national statistics guided the distribution of household sampling by rural-urban (60% vs. 40%), and male-female headed-households (75.7% vs. 24.3%).

Household income also varies considerably between rural and urban areas in Bangladesh. It is postulated that the varied food consumption patterns between rural and urban populations is associated with different socioeconomic and income status. There are also significant differences in the overall livelihoods, occupational composition and lifestyle of the rural and urban populations. Considering these perspectives, the present study was carried out in both rural and urban settings. The Sylhet Division in the northeast region of the country and the capital city of Dhaka South were purposefully selected[i] for rural and urban study sites, respectively.

In order to capture the required diversity, rural sample respondents were selected from three randomly selected *Upazilas* (third-tier administrative unit) of the Sylhet Division, namely Borolekha and Kamolgonj from the Maulovibazar District and Derai from the Sunamganj District. Respondents for the urban component of the study were selected from four Wards (new Ward numbers 1, 11, 40 and 41) of the City Corporation of Dhaka South.

Using the simple random sampling procedure, a total of 501 households were selected, 200 from urban and 301 from rural areas. The distribution of the sampling units (i.e., the households) was determined using the probability proportional to size (PPS) sampling method, i.e., considering the population size of each *Upazila*. Based on the income level and socioeconomic status of each household, a stratified sampling strategy was followed in the final stage of the sampling procedure [22]. The sampling frame, consisting of household income level and socioeconomic status, for each *Upazila* was obtained from the baseline data of previously conducted IDRC project surveys. The specific respondents were then selected randomly from the income and socioeconomic strata in each *Upazila*.

### 2.2. Household Survey

Household survey data were collected from the 301 rural households in Sylhet Division and 200 urban households in the City of Dhaka South as described above. The household head (both males and females) or lead woman of the household was interviewed face-to-face. The survey instrument encompassed identification of the patterns of consumption of fruits and vegetables, expenditures on fruits and vegetables, and economic and non-economic characteristics of the household. This included collecting information on diet over the last 24 h, food frequencies, smoking habits and other health conditions of the respondent and other household members.

### 2.3. Measuring Daily Food Intake

Using a 24 h recall method, participants were asked for information on fruit and vegetable intake during the previous day. Serving size was determined based on the measuring cup recommended by Bangladesh’s national dietary guideline [23]. Accordingly, one serving is equal to: one cup of raw salads or vegetables; a half cup of cooked vegetables or soup; one medium sized piece of raw fruit; and a half cup of chopped fruits or fruit juice. The national standard measurements for serving sizes of fruits and vegetables are shown in Table 1.

### 2.4. Calculation of Belief and Behavioural Practice Index

To analyse the psychosocial aspects of the study, dependent variables were constructed using the additive index of selected pairs of questions from the questionnaire. All questionnaires had a five-point Likert scale response structure (e.g., 5 = strongly agree, 4 = agree, 3 = neutral, 2 = disagree, 1 = strongly disagree). With an additive index, a higher value suggests a higher inclination to agree with the statement. Thus, the index score could range from 1 to 10 as two items were added to construct each index. A total of five output variables (i) food belief, (ii) food status, (iii) subjective belief, (iv) environmental practice and (v) economic practice were calculated to examine which socioeconomic variables help shape these attitudes. The list of the items used to formulate the response variables is shown in Table 2.

### 2.5. Statistical Analysis

The econometric analysis focused on determining the sociodemographic factors affecting both social food beliefs and behavioural practices, and low intake of fruits and vegetables. To examine the first, whether and how did social beliefs and behavioural practices affect diet were assessed using an ordinal logistic regression, where the dependent variables had an ordered score ranging from 10 to 1. For the second analytical focus, a binary logistic regression analysis was performed to test the hypothesis concerning the influence of sociodemographic variables on inadequate fruit and vegetable consumption. For this, the dependent variable was constructed as 1 = consumed less than 5 serving of fruits and vegetables per day and 0 = consumed equal to or more than 5 servings per day. Age, level of education, type of occupation, household monthly income, marital status, gender, residence, the habit of eating out and smoking were all examined in relation to participants’ low intake of fruits and vegetables. Multicollinearity was tested prior to the final regression using a correlation matrix among the predictors. Odds ratios (OR) were computed with a confidence interval of 95%. All analysis were performed using the software STATA version 13.

## 3. Results

### 3.1. Socioeconomic Description of the Respondents 

Table 3 presents summary statistics on the sociodemographic conditions of the two study areas. Of the 501 heads of household (HHs) interviewed, 301 (60%) were from rural areas and 200 (40%) were from urban settings. In rural areas, 81% (*n* = 301) of the participants were male. There were more female respondent HHs in the urban (32%) compared to the rural areas (19%). The mean age of respondents (for both male and female HHs) was 48 years in rural areas. However, in urban areas, the mean age for women was higher (53 years) than in rural areas (48 years).

In rural areas, the majority (60%) of the female HHs were divorced or widowed; in urban areas, it was 33% of the female HHs. Of the male participants, 98% were married. In rural areas, more than 50% of the participants had no formal schooling. Only 2% of the male participants had completed higher secondary or tertiary education, none of the female participants completed higher secondary level. In urban areas, more than 50% of the participants had primary or secondary education. Notably, more than one-third of both male and female participants had either higher secondary or tertiary level of education.

The main occupations of the rural men were day labour (33%), agricultural work (30%) and self-employed (12%). Most rural women identified themselves as housewives (64%). In urban areas, 46% of the men and 25% of the women worked in the business sector, and 28% of the men and 20% of the women worked in the service sector.

The majority of the households (>81%) in rural areas earned less than BDT 15,000 (US $177.5) per month, and only 2.8% earned more than 30,000 BDT (US $355) per month. However, 49% of the participants living in urban areas earned more than 30,000 BDT (US $355) per month.

Given that the mean age was over 48 years, very few participants had children less than 5 years of age. Comparing everyday lifestyle, tobacco use was much higher in rural areas compared to urban areas; 65% of rural males and 26% urban males smoked. Among the female participants, 16% in rural areas compared to 6.3% urban areas smoked. As well, overwhelming majority (88%) of rural female participants chewed tobacco. Noticeably, urban male (40%) and female (21%) participants reported more physical activity than rural participants (only 3%).

### 3.2. Distribution of Low Daily Fruit and Vegetable Intake

The data regarding the servings of fruits and vegetables the respondents had eaten in the previous day are shown in Table 4, and are presented as the percentage of participants in each sociodemographic category who consumed less than and more than the recommended five servings per day. Approximately 92% of participants in rural areas and 75% in urban areas had consumed less than 5 servings of fruits and vegetables over the previous 24 h. This reveals that participants from urban areas were likely to eat more fruits and vegetables than the rural people.

A pattern of consuming more fruits and vegetables was observed with middle-aged (35–55 years old) people, while a decreasing trend was found among people aged over 65 and those younger than 35. Women in the rural areas ate considerably less fruits and vegetables than men, whereas there was no noticeable difference in intake between urban males and females.

Education was found to be a good predictor for intake of more fruits and vegetables. People with secondary, higher secondary and tertiary education were likely to eat more fruits and vegetables in their everyday meals. An increase in earnings was also associated with a higher intake of fruits and vegetables, in both urban and rural areas. Having children less than five years of age in the household also increased the probability of eating more than 5 servings of fruits and vegetables in both areas.

In rural areas, percentage of participants who ate more than 5 servings a day was slightly higher among those who smoked cigarettes (8.3%) compared to no smoking (7.3%), chewed tobacco (8.5%) compared to no tobacco (7.0%) and did no physical exercise (8.2%) compared to regular physical activities (0.0%), unlike their urban counterparts. However, people who ate at the end of family eating sessions and those who bought fast food regularly were less likely to consume more than 5 serving of fruits and vegetables.

### 3.3. Descriptive Analysis of Respondents’ Social Belief and Behavioural Practice in Terms of Fruits and Vegetable Consumption

Bivariate and descriptive analyses of the respondents’ beliefs and behavioural practices are shown in Table 5. When they were asked if rice was more important than vegetables in everyday meals, the majority of the respondents (73.8%) agreed and only a few (25.6%) disagreed. However, most people (64%) disagreed with the statement that meat was more important than vegetables, which means that, among the three food categories, rice was regarded the most important. A smaller majority of respondents also disagreed with the popular beliefs that vegetables are food for the poor (57.6%), and that fish and meat are food for the rich (51.8%). When respondents were asked whether they think their families eat enough fruits and vegetables, the majority of the participants (>64%) indicated they did not.

A chi-square test revealed that significant differences existed regarding social food beliefs among respondents in different socioeconomic status or income groups. For example, 85% of participants who earned less than 15,000 BDT (US $177) agreed that rice is more important than vegetables, whereas only 44.6% of the higher income group agreed with the statement. Similarly, 58.9% of participants in the low-income group, 21.6% of the middle-income group and only 10.9% from the high-income group agreed that vegetables are food for the poor. People who believed that ‘meat and fish are food are mainly for the rich’ mostly (65.1%) were from the low-earning groups; such beliefs had lesser effects (10%) up on the high income group. Thus, households with lower income were more likely to agree with popular food-related beliefs that are prejudiced against a higher consumption of fruits and vegetables. Contrary to prejudiced food belief, people with higher income (53%) were more agreeable to the statement that their families eat enough fruits than the lower income group (19%). This is also indicative that higher income status may influence a higher consumption of fruits and vegetables.

Regarding behavioural practices, 62.3% of the respondents agreed and 30.9% disagreed when asked whether formalin is more important than high prices in deterring them from buying more fruits. However, pesticide use on vegetables seemed less alarming for the participants only 38% agreed that pesticide use was a more important factor than high price when buying vegetables. In the case of increased income, respondents indicated that they would be more likely to buy more meat (57.8%) or rice (43.9%) instead of more vegetables.

Significant differences in social practices existed among respondents from different socioeconomic status or income groups (*p*-values: 0.0 to 0.001). The distribution of social practices with social status showed a similar pattern to what was found for social beliefs, i.e., richer respondents were more likely to disregard prevalent social practices. A total of 73.5% of respondents with lower social status agreed that with an increase in income they would buy more meat whereas only 23.9% from higher status agreed to the statement. Similarly, with an income rise, 58.6% from the lower earning status would buy more rice than vegetables compared to only 18.5% among the higher income group.

**Figure 1:**
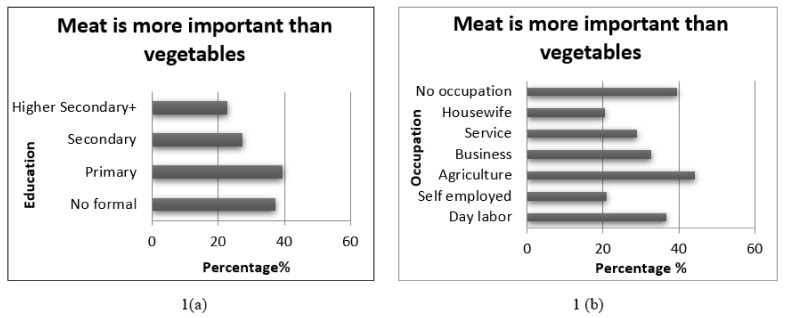
Distribution of responses to the statement ‘meat is preferable to vegetables’ by educational attainment (**a**) and occupational category (**b**).

In order to analyse the underpinnings of responses to the statement ‘meat is more important than vegetables’, the distribution by educational attainment and occupation was examined. As Figure 1a illustrates, with some level of education, people were less likely to comply with popular, prejudiced beliefs regarding fruit and vegetable intake. Figure 1b further reveals that people who were unemployed or work in the agriculture sector were more likely to subscribe to the idea that ‘meat is more important than vegetables for health’.

**Figure 2:**
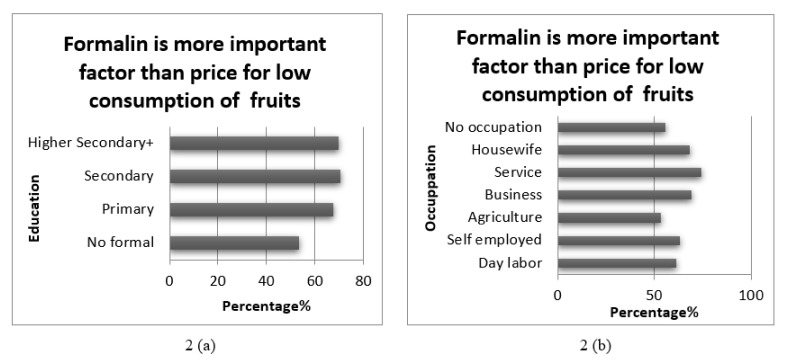
Distribution of responses to the statement ‘Formalin is a more important factor than price for low consumption of fruits’ by level of education (**a**) and occupational categories (**b**).

On the contrary, patterns in Figure 2a,b indicate that the more highly educated compared to ‘no formal education’ and those in the service sector compared to agriculture were more concerned about formalin on fruits and vegetables over price. Service holders were likely to have higher education background, higher income status, and easier and more access to resources (TV, radio, health worker) to know about food safety than a farmer or a day-labourer.

### 3.4. Econometric Analysis of Social Beliefs and Social Practices in Terms of Sociodemographic and Lifestyle Variables

Ordinal logistic regressions were performed to assess which socioeconomic variables had more influence on shaping popular beliefs and behavioural practices regarding fruit and vegetable consumption, and the results are presented in Table 6. Model 1 revealed that respondents who had higher secondary education compared to those with no education/schooling were less likely to agree with the belief that ‘rice or meat is more important than vegetables’. However, people living in rural areas, businessmen and those who regularly ate processed food were more likely to accept these popular beliefs. Highly educated people seemed to disapprove such beliefs.

In Model 2, respondents who earned medium or higher income in comparison to low income, businessmen and those who had some level of education in comparison with no education background were all less likely to agree that ‘vegetables are food for the poor, and fish and meat are food for the rich’. Respondents living in rural areas who ate processed food were more likely to agree with this idea. In explaining Model 2, higher education and income were found to be most significant.

Model 3 explored the subjective beliefs of respondents regarding the volume (how much) of fruits and vegetables their family eats. Respondents who were female, working in agriculture, with lowest (primary) or highest (bachelor) level of educational backgrounds, and those who ate fast foods regularly (except for those who lived in rural areas), were all less likely to agree that ‘their family eats enough fruits and vegetables’. Income seemed to have no effect on Model 3.

Model 4 showed that respondents who had primary or secondary education, those who exercise regularly, who worked in the service sector with medium income and those living in rural areas were more likely to agree that ‘formalin or pesticide use is a far more important issue to consider than price before buying fruits and vegetables’. No significant disagreement found in this model.

In model 5, it was seen that respondents with the highest education, medium to high income, those married with children under five and respondents from the oldest and youngest age cohorts were less likely to agree with the popular statement that ‘when income rises more rice or meat is bought’. However, people from rural areas who consumed smokeless tobacco, those who bought processed food, who were self-employed and those who worked in the service sector were all more likely to think otherwise.

### 3.5. Econometric Analysis of Low Intake of Fruits and Vegetable in Terms of Sociodemographic and Lifestyle Variables

Table 7 presents the results of a logistic regression concerning the association between various sociodemographic and lifestyle factors and low fruit and vegetable intake. Old age, higher education and higher income were found to be associated with a lower probability of a low intake of fruits and vegetables. People in the 55–64 age group were 68% less likely than those in the 25–34 age group to consume low levels of fruits and vegetables. Those with higher education, especially secondary (74%) and bachelor’s degrees (71%), were less likely to eat lower amount of fruits and vegetables in their daily meals. Respondents with medium and high earnings were 43% and 69%, respectively, less likely to be at risk of low fruit and vegetable consumption. No industrial occupation seemed to have had any significant effect on low intake of fruits and vegetables except being housewives.

Respondents who were housewives, those who ate processed food and those eating at the end of family serving sessions were respectively 69%, 48% and 27% more likely to be at risk of eating too few fruits and vegetables, and this pattern was statistically significant. Place of residence (rural vs. urban), sex (male vs. female), having children (under five years vs. no child) and other lifestyle features (e.g., smoking, chewing tobacco, exercise) did not have any significant effects.

## 4. Discussion

Results of our investigation reveal that approximately 92% of rural respondents and 75% in urban areas consume less than the recommended five servings of fruits and vegetables every day in Bangladesh. This result is consistent with many other LMICs, such as Mexico and Thailand, where fruits and vegetables are produced mainly to export to other countries [24]. Participants aged 35–55 were more likely to eat more servings of fruits and vegetables than the youngest (25–34) and oldest (65+) age groups. Although the 18–25 age group is most often found to be the most vulnerable group for low intake of fruits and vegetables [23], in our study both the young and elderly groups were more vulnerable. The prevalence of a low intake of fruits and vegetables generally decreases with age in high-income countries like the USA and France; however, one study also showed that in the USA this holds true among whites and Hispanics but not among the African American population [22]. In LMICs, different age-related patterns are likely to appear due to their varied demographic age structure and dynamics. Further research on these aspects is required.

Like most of the existing studies, higher education and a higher income were found to boost the intake of fruits and vegetables in both rural and urban areas [25]. The higher price of fruits and vegetables is a usual barrier for low-income people to consume them, even in high income, developed countries. The fact that a higher fruit and vegetable intake is associated with higher education is indicative that those respondents make an informed decision when buying food. However, in some countries, food culture is a more prevalent factor than mere affordability or education level.

In general, people with unhealthy lifestyles are less interested in eating fruits and vegetables. In our study, contrary to this, respondents in rural areas who smoked tobacco or were not engaged in physical activity were found to eat more servings of fruits and vegetables than those who had a healthier lifestyle. Buying cigarettes or chewing tobacco is expensive for most poor, rural people who cannot afford three meals per day. Moreover, rural people usually walk more than urban people because of deficiencies in transportation facilities within the area, and also stores are usually located far from the households. These findings indicate that the rural respondents who consumed tobacco products were most likely from the higher income groups who could afford both recreational substances and convenient modes of transportation, as well as fruits and vegetables.

Findings about social beliefs and behavioural practices regarding fruit and vegetable consumption in relation to socioeconomic status revealed that people with lower income were more likely to believe in food beliefs and behavioural practices prejudiced against higher levels of fruit and vegetable consumption. The only exception was when participants were asked whether they thought their family ate enough fruits or vegetables, and if formalin was the reason why they did not prefer fruits. As expected, participants with higher incomes and higher levels of education were more likely to agree with these statements. It is apparent that participants were more afraid of formalin than they were of pesticide use in vegetables. Only 38% of participants agreed that ‘pesticides used in vegetables act as an excluding criteria while selecting vegetables for grocery’, whereas 62.3% thought formalin is a bigger impediment. With more income and education, people became more likely to regard formalin as a deterrent for buying fruits, but pesticide use in vegetables followed an opposite trend where with more income and education people were more accepting of vegetables with pesticides.

The fear of formalin in fruits among the Bangladeshi people started during 2005 [26]. Public discontent over basic food safety and the ensuing political crisis exerted pressure on the government to take quick action on food safety. Consequently, a mobile food court system emerged as a solution, and from May 2005 to October 2006 a total of 2139 mobile court operations were conducted to combat food adulteration [26]. The courts gathered US $13,971 in fines, filed over 16,000 cases and imprisoned 782 persons. After these punitive and regulatory measures by the government, people became more afraid of formalin than any other form of food adulteration.

Most respondents (especially low-income people) believed that ‘rice is more important than vegetables in everyday meals’ but fewer people agreed that ‘meat is more important than vegetables’. This implies that rice is the main staple of Bengali peoples’ everyday meals. Meat is not easily affordable for poor people in both cities and rural villages. This finding corroborates the lesser importance of meat compared to rice. However, when the same participants were asked whether they would buy more meat or rice when income was increased, they agreed with the statement that they would buy a higher amount of meat (57%) than rice (43.9%).

Econometric analysis of the five summative belief (food belief, food status, subjective belief) and behavioural practice (environmental and economic) indices in our study showed that the most significant factors shaping food attitudes were respondents’ place of residence, level of education, household income, occupation and access to processed fast food. Age, sex, marital status and lifestyle choices had limited significance and little influence on the indices.

Prevalent social food beliefs promote the notion that ‘rice or meat is more important than vegetables’ in Bangladesh’s food culture. Respondents with higher secondary education were less likely to agree with this statement because they were more aware of the benefits of fruit and vegetable intake. Many of the previous studies similarly found that with higher education fruit and vegetable intake increases [27,28,29]. In our study, rural people, businessmen and respondents with access to fast food were more likely to hold this belief regarding the lesser importance of fruits and vegetables. Most rural people in the study area had little to no education and lacked adequate nutritional knowledge, and therefore tended to agree with the statement. Similarly, respondents who favoured fast food or who had no option but to eat from roadside eateries appeared to care more about saving money and time than about nutrition [30]. Small businessmen did not always have much institutional education, and so the finding regarding them supports the association between education level and social food beliefs.

All independent variables functioned similarly for the food status index as they did for the food belief index, with three exceptions. Higher education, higher household income and being businessmen were all associated with disagreeing with the food status index statements regarding fruits and vegetables being food for the poor and fish and meat for the rich. In the previous food belief index, businessmen, unlike the highly educated respondents, were more likely to believe rice and meat are more important than vegetables, indicating a lack of nutritional knowledge. In respect of the food status question, both businessmen and the more highly educated respondents were more likely to disagree with the statement. The most likely reason for this disagreement by businessmen was that they no longer belong to the poor socioeconomic class or empathise with the poor. This is similar to Veblen’s theory of conspicuous consumption where the wealthy buy luxury goods to signal social status [31]. In this case we posit that businessmen were mirroring the response of the more nutritionally educated respondents to uphold their higher status.

Both the least educated and the most educated respondents were found less likely to report that they eat enough fruits and vegetables. The probable explanation for the least educated group is related to their low income and the affordability of healthy food every day. Presumably the most educated group know more about nutrition and so were more aware that they were not taking enough servings of fruits and vegetables every day. Being female was found significant only in regard to this statement, which might indicate that females were more often deprived of appropriate amounts of fruits and vegetables, or that, as they were the ones who cook, they knew better than male respondents whether the family eats enough fruits and vegetables.

Environmental practice was the only index where all independent variables were positively related to the index. Attainment of primary and secondary education, being in the service sector and regular physical fitness were all associated with agreeing that concern about formalin and pesticide use were more important factors than price in driving low consumption of fruits and vegetables. Primary and secondary schools in the country always offer educational courses on basic food hygiene (washing hand) to food safety awareness (chemical residue on food) to avoid major food borne diseases. Additionally, several formalin incidents in the country changed the overall consumer demand for safe food however, not so much for nutritious food. This shift in demand and rise in health conscious (physical fitness) influenced people to be more environmentally conscious, which is well reflected in this index. In comparison to day-labour, people who work in the service sector were more likely to be more educated, richer and have had easier access to food safety education that would influence them to put more emphasis on safer food than price. However, contrary to our findings, in some previous studies, sex of the consumer was significant. Female respondents were found to be more environmental and health conscious than males [32].

The regression analysis for predicting low fruit and vegetable intake showed that respondents with higher education, higher income, and from the older age cohorts were at the least risk of low intake of fruits and vegetables. This result is consistent with several other studies [32]. Globally, low income is the strongest predictor of insufficient fruit and vegetable intake, and education beyond the secondary level is a key variable in reaching the recommended level of consumption [33]. In our study, older people had lower odds of inadequate consumption compared to the young adults (25–35 age), which is contrary to the findings of a previous WHO-STEPS study [27]. One explanation for this difference may lie in the age composition of the WHO-STEPS study, where the majority of the respondents were from the younger (25–34) age group, while in our study the majority of the respondents were from the middle to older age groups.

Moreover, unlike the findings of the WHO STEP study, in our study the sex of the respondents did not affect inadequate consumption [27], although we found that the occupational category of housewives was more likely at a higher risk of low intake. A study conducted in five Asian countries found that women were more likely to be at risk of inadequate fruit and vegetable intake in India and Bangladesh, but less likely in some other Southeast Asian countries, namely Vietnam, Indonesia and Thailand [29]. However, interestingly, in high-income countries women were found to consume more fruits and vegetables than men [34,35]. The main reason for such a pattern is that women in high-income countries are generally more health aware and willing to maintain a healthy diet.

Although the location of residence (i.e., rural or urban) did not appear to be significant in the logistic regression, the descriptive analysis showed a major difference in fruit and vegetable intake. On average, 25% of urban males and females consumed more than five servings each day, whereas it was less than 9% in rural areas. A study in 52 countries found that overall urbanity had no association with low intake of fruits and vegetables, with a difference noted in 11 countries [28]. On the contrary, urban people in sub-Saharan African consumed a higher amount of fruits and vegetables than their rural counterparts [12,25]. With globalisation, since the 1990s eating habits in Asian countries have shifted from traditional diets (i.e., cereal and low fat-based) to a more Western diet (i.e., more meats, animal fats and refined sugar), primarily due to new international food supply chains and the opening of supermarkets [12,36,37]. These forces have enabled greater access to and affordability of a variety of fruits and vegetables for urban dwellers. However, this access and affordability has remained very limited in rural areas [29,38]. Availability has emerged as a major constraint because most farmers produce chiefly for export and not for the local market [39,40].

There were several limitations to our study. We used the 24 h recall method to collect data on consumption, and this information was entirely dependent on respondents’ own capacity and memory. This self-reported data may result in some over or underestimation of the data. Moreover, in our investigation, we did not capture the aspect of seasonal variations, nor did we achieve a national level of representation. Different social, cultural and geographic factors, as well as variations in lifestyle, habits, nutritional knowledge or cooking styles may also affect the level of consumption [28], and these were not addressed in the present study. Further research should be carried out to examine these potential explanatory factors for low fruit and vegetable consumption.

## 5. Conclusions

The objective of this study was to identify the socioeconomic and sociodemographic factors that influence the low intake of fruits and vegetables in Bangladesh, and determine the factors associated with maintaining popular social beliefs and behavioural practices related to prejudices against fruit and vegetable consumption. Both rural and urban residents were studied to gain comparative insights regarding factors that are correlated with fruit and vegetable consumption.

The findings of our study revealed that, on average, 75–92% of the population of Bangladesh do not consume five servings of fruits and vegetables a day, and this is applicable to both urban and rural settings. Gender was not generally found to play any significant role in shaping attitudes or consumption. However, women who are by occupation housewives were more at risk to low consumption of fruits and vegetables. Urban respondents reported eating slightly more servings per day than rural people did.

The location of residence and access to fast food shape social beliefs and behavioural practices significantly. Having a higher income tended to decrease the risk of lower consumption of fruits and vegetables, but did not always help with developing attitudes in favour of healthy food choices. Higher educational attainment was the sole measure associated with achieving both the recommended goal for fruit and vegetable consumption and non-prejudiced attitudes towards their consumption.

Finally, it is recommended that in order to promote adequate fruit and vegetable consumption, policymakers should establish interventions sensitive to placed-based contexts and regional (rural-urban), cultural and psychosocial factors that affect consumer choices and preferences. Governmental policy-formulating agencies need to focus on increasing opportunities for mass education, particularly for females. In this regard, the national budget should allocate more priority to public education and wage-earning job training programs for women. In addition, taking lessons learned from the mass media campaign about formalin concerns, NGOs should also promote Behaviour Change Communications on the benefits of fruit and vegetable consumption. Monitoring food safety and the nutritional quality of local fast food stores, and undertaking appropriate regulatory measures by relevant governmental departments are also necessary to address the low intake of fruits and vegetables in Bangladesh.

## Figures and Tables

**Table 1:** Standardised measurements for fruits and vegetable intake (showcard for serving size).

Items	Portion Size = 1 Serving	Gm Per Serving	Example
**Vegetables**	1/2 Cup	100	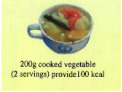
Cooked vegetables			
Soup/juice of vegetables	1/2 Cup	80	Lettuce, cucumber 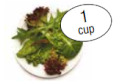
Raw salad/vegetables	1 Cup	80	
**Fruits**	1 medium size	100	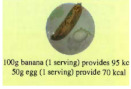
Apple/banana/orange			
Small fruits	2–8 pieces	100	8 Jujubes, 2 plums
Chopped/canned fruit	1/2 cup	80	Watermelon
Fruit juice	1/2 cup	80	Mango, Orange

Source: National dietary guideline for Bangladesh BIRDEM, 2013[23].

**Table 2:** Index of social beliefs and behavioural practices index.

Additive Index	Items
Food belief	‘Rice is more important than vegetables in everyday meals’
	‘Meat is more important than vegetables in daily meals’
Food status	‘Vegetables are food for the poor’
	‘Fish and meat are food for the rich’
Subjective belief	‘My family members eat enough vegetables’
	‘My family members eat enough fruits’
Environmental practice	‘Formalin on fruits is a more important reason than higher price for low intake of fruits’
	‘Pesticide use on vegetables is a more important driver for low intake of vegetables’
Economic Practice	‘If income rises, more meat is purchased than vegetables’
	‘If income rises, more rice is purchased than vegetables’

**Table 3:** Sociodemographic characteristics of the HH survey participants.

Variables	Rural (*n* = 301)		Urban (*n* = 200)	
	Male	Female	Male	Female
*n*	243 (81%)	58 (19%)	136 (68%)	64 (32%)
Mean Age	48.8	48	48.3	53
Age Group				
25–34	15.6	17.2	17.7	4.7
35–44	23.5	15.5	22.8	17.2
45–55	23.9	32.8	23.5	28.1
55–64	20.2	25.9	20.6	29.7
65+	16.9	8.6	15.4	20.3
Marital Status				
Married	98.4	39.7	97.8	67.2
Divorced/Separated/Widowed	1.7	60.3	2.2	32.8
Religion				
Muslim	62.6	82.8	87.5	85.9
Hindu	37.5	17.2	12.5	14.1
Education				
No formal	52.3	65.5	11.8	9.4
Primary	32.5	31.0	24.3	26.6
Secondary	13.2	3.5	25.7	29.7
Higher Secondary+	2.1	0.0	38.2	34.4
Occupation				
Day labour	33.3	5.2	5.2	10.9
Self employed	11.9	1.7	4.4	3.1
Agriculture	30.5	5.2	-	-
Business	9.1	5.2	46.3	25.0
Service	3.3	-	27.9	20.3
Housewife	-	63.8	-	10.9
No occupation	11.9	19.0	16.2	29.7
HH Income/month (BDT)				
<15000	82.4	79.3	22.3	11.5
15001–30000	13.9	19.0	36.4	26.9
>30000	3.8	1.7	41.3	61.5
Number of Children < 5				
0	53.5	69.0	61.0	68.8
1	26.3	17.2	30.9	14.1
2+	20.2	13.8	8.1	17.2
Smokes tobacco				
No	34.6	84.5	74.3	93.8
yes	65.4	15.5	25.7	6.3
Smokeless Tobacco				
No	38.3	12.1	81.6	71.9
Yes	61.7	87.9	18.4	28.1
Physical Activity				
No	96.7	96.6	78.7	85.9
Yes	3.3	3.5	21.3	14.1

Source: Field surveys by the first author in 2019.

**Table 4:** Descriptive and bivariate χ^2^ analysis of sociodemographic variables according to participants’ per day intake of fruits and vegetables.

Variables	Rural (*n* = 301)		Urban (*n* = 200)		*p*-Value
	<5 Serving	≥5 Serving	<5 Serving	≥5 Serving	
**n** **Age Group+**	92.03	7.97	75.00	25.00	
25–34	93.75	6.25	85.19	14.81	0.2973
35–44	90.91	9.09	69.05	30.95	
45–55	90.91	9.09	74.0	26.0	
55–64	92.73	7.27	75.31	24.69	
**Sex**					0.9953
Male	91.0	9.1	75.0	25.0	
Female	96.6	3.5	75.0	25.0	
**Marital Status**					
Married	91.98	8.02	73.86	26.14	0.3822
Divorced/Separated	92.31	7.69	83.33	16.67	
**Religion**					
Muslim	92.00	8.00	75.29	24.71	0.2776
Hindu	92.08	7.92	73.08	26.92	
**Education**					
No formal	94.55	5.45	90.91	9.09	0.0000 *
Primary	93.81	6.19	84.0	16.0	
Secondary	76.47	23.53	72.22	27.78	
Higher Secondary+	80.0	20.0	66.22	33.78	
**Occupation**					
Day labour	94.05	5.95	85.71	14.29	0.0000 *
Self employed	86.67	13.33	100.00	0.00	
Agriculture	90.91	9.09			
Business	92.00	8.00	65.82	34.18	
Service	75.00	25.00	78.43	21.57	
Housewife	100.00	0.00	85.71	14.29	
No occupation	90.00	10.00	78.05	21.95	
**HH Income/month (BDT)**					
Low: <15.000	94.21	5.79	87.88	12.12	0.0000 *
Medium: 15.001–30.000	86.36	13.64	72.41	27.59	
High: >30.000	70.0	30.0	68.29	31.71	
**Number of Children**					
0	92.35	7.65	77.17	22.83	0.9514
1	91.89	8.11	72.55	27.45	
2+	91.23	8.77	68.18	31.82	
**Smokes tobacco**					
No	92.48	7.52	72.05	27.95	0.0033 *
Yes	91.67	8.33	87.18	12.82	
**Smokeless Tobacco**					
No	93.0	7.0	71.97	28.03	0.0011 *
Yes	91.54	8.46	86.05	13.95	
**Physical Activity**					
No	91.75	8.25	74.69	25.31	0.4147
Yes	100	0	76.32	23.68	
**Eat at the end**					
No	85.23	14.77	62.35	37.65	0.0049 *
Yes	97.22	2.78	78.13	21.88	
**Eat fast food regularly**					
No	89.94	10.06	67.83	32.17	0.0016 *
Yes	94.37	5.63	84.71	15.29	

* Significant at *p* < 0.05 level. + These bolded headings are showing the broad categories of variables.

**Table 5:** Descriptive and bivariate χ^2^ analysis of the responses to the popular social beliefs and behavioural practices according to socioeconomic status.

Attitudes	Statements	Response	%	HH Monthly Income			*p*-Value
				Low	Medium	High	
Food belief	‘Rice is more important than vegetables in everyday meals’	Disagree	25.6	13.8	31.4	54.4	0.0000 *
		Neutral	0.6	0.7	0.0	1.1	
		Agree	73.8	85.5	68.6	44.6	
	‘Meat is more important than vegetables in daily meals’	Disagree	64.0	58.9	73.5	68.5	0.0959
		Neutral	1.3	1.5	1.0	1.1	
		Agree	34.8	39.6	25.5	30.4	
Food status	‘Vegetables are food for the poor’	Disagree	57.6	39.3	78.4	89.1	0.0000 *
		Neutral	1.1	1.8	0.0	0.0	
		Agree	41.4	58.9	21.6	10.9	
	‘Fish and meat are food for the rich’	Disagree	51.8	32.7	75.5	82.6	0.0000 *
		Neutral	1.7	2.2	2.0	0.0	
		Agree	46.5	65.1	22.5	17.4	
Subjective belief	‘My family members eat enough vegetables’	Agree	32.2	24.0	38.2	50.0	0.0000 *
		Neutral	6.0	9.1	2.9	0.0	
		Disagree	61.8	66.9	58.8	50.0	
	‘My family members eat enough fruits’	Agree	26.6	19.3	29.4	45.6	0.0000 *
		Neutral	6.4	9.1	3.9	1.1	
		Disagree	67.0	71.6	66.7	53.3	
Environmental practice	‘Formalin on fruits are a more important reason than higher price for why fruits are eaten less’	Disagree	30.9	33.1	22.5	33.7	0.0003 *
		Neutral	6.8	10.5	2.9	0.0	
		Agree	62.3	56.4	74.5	66.3	
	‘Pesticides on vegetables are a more important driver of eating less vegetables even if the price is low’	Disagree	54.2	47.3	57.8	70.6	0.0012 *
		Neutral	7.9	10.5	5.9	2.2	
		Agree	38.0	42.2	36.3	27.2	
Economic practice	‘If income rises more meat is bought than vegetables’	Disagree	38.6	21.8	51.0	75.0	0.0000 *
		Neutral	3.6	4.7	2.9	1.1	
		Agree	57.8	73.5	46.1	23.9	
	‘If income rises more rice is bought than vegetables’	Disagree	50.8	34.9	66.7	80.4	0.0000 *
		Neutral	5.3	6.5	5.9	1.1	
		Agree	43.9	58.6	27.5	18.5	

* Significant at *p* < 0.05 level.

**Table 6:** Ordered logistic regression of popular social beliefs and behavioural practices (odds ratios).

Variables	(1)	(2)	(3)	(4)	(5)
	OR of Food Belief	OR of Food Status	OR of Subjective Belief	OR of Environmental Practice	OR of Economic Practice
**Age Group ^a +^** 35–44 years	0.947	0.719	0.762	1.007	0.503 **
	(0.272)	(0.229)	(0.225)	(0.282)	(0.151)
45–54	0.775	0.713	1.193	1.146	0.627
	(0.221)	(0.230)	(0.356)	(0.330)	(0.192)
55–64	1.031	0.859	0.956	1.205	0.740
	(0.310)	(0.287)	(0.298)	(0.353)	(0.234)
65+	0.979	0.844	0.633	1.240	0.405 ***
**Education ^b^**	(0.326)	(0.322)	(0.215)	(0.406)	(0.141)
Primary education	1.149	0.630 **	0.609 **	1.460 *	0.876
	(0.249)	(0.142)	(0.135)	(0.308)	(0.191)
Secondary education	0.652	0.514 **	0.720	1.578 *	0.661
	(0.186)	(0.151)	(0.210)	(0.427)	(0.185)
Higher Secondary+	0.448 **	0.245 ***	0.357 ***	1.337	0.207 ***
**Occupation ^c^**	(0.163)	(0.110)	(0.132)	(0.468)	(0.0848)
Self employed	0.780	0.779	0.709	1.348	2.346 **
	(0.278)	(0.295)	(0.266)	(0.467)	(0.914)
Agriculture	1.084	0.647	0.418 ***	0.653	1.192
	(0.314)	(0.199)	(0.128)	(0.193)	(0.345)
Business	1.975 **	0.385 ***	0.767	1.568	0.707
	(0.629)	(0.131)	(0.247)	(0.485)	(0.231)
Service	1.863	0.890	0.863	2.049 *	1.968 *
	(0.719)	(0.367)	(0.340)	(0.775)	(0.784)
Housewife	0.866	0.502	0.863	0.983	0.782
	(0.359)	(0.232)	(0.370)	(0.406)	(0.340)
No occupation	1.502	0.859	0.785	0.708	0.999
**Income/month (BDT) ^d^**	(0.504)	(0.309)	(0.272)	(0.237)	(0.343)
15000–30000	0.876	0.347 ***	0.933	1.486 *	0.648 *
	(0.216)	(0.0881)	(0.229)	(0.352)	(0.161)
>30000	0.855	0.251 ***	0.813	1.150	0.564 *
	(0.257)	(0.0872)	(0.256)	(0.338)	(0.187)
**Married** (Marital Status)	1.205	1.033	0.660	1.229	0.437 **
	(0.390)	(0.355)	(0.218)	(0.385)	(0.151)
**Rural** (Residence)	7.602 ***	3.189 ***	1.995 **	5.188 ***	6.812 ***
	(2.348)	(0.957)	(0.584)	(1.496)	(2.116)
Female (Sex)	0.731	0.910	0.600 *	1.051	1.479
	(0.207)	(0.319)	(0.175)	(0.292)	(0.470)
**Presence of children < 5**	0.956	1.023	0.993	1.008	0.803 *
	(0.107)	(0.123)	(0.115)	(0.112)	(0.0933)
**Smokes cigarettes**	1.077	0.794	1.121	0.948	0.905
	(0.212)	(0.169)	(0.223)	(0.178)	(0.183)
**Chews tobacco**	1.305	1.407	1.165	0.924	1.552**
	(0.257)	(0.294)	(0.238)	(0.179)	(0.309)
**Physical activity**	1.430	0.836	1.607	2.104 **	0.724
	(0.476)	(0.328)	(0.526)	(0.662)	(0.273)
**Eat fast food**	1.405 **	1.617 **	0.726 *	0.794	1.349 *
	(0.242)	(0.314)	(0.130)	(0.135)	(0.244)
Constant cut1	0.394 **	0.254 ***	0.0395 ***	0.440 *	0.276 ***
	(0.172)	(0.116)	(0.0188)	(0.195)	(0.127)
Constant cut2	0.637	0.295 ***	0.0641 ***	0.945	0.366 **
	(0.275)	(0.134)	(0.0297)	(0.414)	(0.167)
Constant cut3	0.907	0.660	0.113 ***	1.601	0.637
	(0.390)	(0.298)	(0.0514)	(0.698)	(0.288)
Constant cut4	1.127	0.727	0.151 ***	2.427 **	0.758
	(0.483)	(0.328)	(0.0680)	(1.057)	(0.342)
Constant cut5	4.088 ***	0.976	0.411 **	6.178 ***	2.138 *
	(1.768)	(0.440)	(0.183)	(2.725)	(0.965)
Constant cut6	13.58 ***	1.337	0.545	8.504 ***	3.539 ***
	(6.033)	(0.603)	(0.242)	(3.780)	(1.606)
Constant cut7	14.57 ***	1.961	0.697	13.39 ***	4.852 ***
	(6.485)	(0.884)	(0.309)	(6.026)	(2.210)
Constant cut8	23.95 ***	2.414 *	0.945	20.21 ***	9.386 ***
	(10.81)	(1.089)	(0.419)	(9.183)	(4.325)
					
Observations	469	469	469	469	469
Pseudo R-squared	0.0914	0.150	0.0454	0.0266	0.143

^a^ Reference age: 25–34; ^b^ reference education: no education; ^c^ reference occupation: day-labour; ^d^ reference earning: less than 15,000 BDT; exponentiated coefficients; *t* statistics in parentheses; *** *p* < 0.01, ** *p* < 0.05, * *p* < 0.1. + These bolded headings are showing the broad categories of variables.

**Table 7:** Logistic regression of daily low fruit and vegetable consumption.

Variables	<5 Serving of Fruits and Vegetable Intake
**Age group ^a +^** 35–44 years	0.400
	(0.228)
45–54	0.422
	(0.244)
55–64	0.322 *
	(0.190)
65+	0.625
**Education ^b^**	(0.424)
Primary	0.721
	(0.348)
Secondary	0.265 ***
	(0.135)
Higher Secondary+	0.290 **
	(0.173)
**Occupation ^c^** Self employed	1.269
	(1.003)
Agriculture	0.970
	(0.608)
Business	0.616
	(0.352)
Service	1.141
	(0.753)
Housewife	10.31 *
	(12.61)
Unemployed	1.285
	(0.830)
**Income/month (BDT) ^d^** 15000–30000	0.434 *
	(0.193)
>30000	0.314 **
	(0.160)
**Married**	0.448
	(0.293)
**Rural**	0.832
	(0.411)
**Female**	1.276
	(0.554)
**Presence of children <5**	0.797
	(0.163)
**Smokes cigarettes**	1.452
	(0.519)
**Chews tobacco**	1.065
	(0.393)
**Physical activity**	1.666
	(0.836)
**Eats fast food**	2.517 ***
	(0.839)
**Eat at the end**	2.773 ***
	(1.080)
Constant	23.84 ***
	(19.34)
	
Observations	469
Pseudo R-squared	0.216

Exponentiated coefficients; *t* statistics are within parentheses; *** *p* < 0.01, ** *p* < 0.05, * *p* < 0.1; ^a^ reference age: 25–34; ^b^ reference education: no education; ^c^ reference occupation: day-labour; ^d^ reference earning: less than 15,000 BDT. + These bolded headings are showing the broad categories of variables.

## Data Availability

The datasets used and/or analysed during the current study are available from the corresponding author on reasonable request.
